# Geographical accessibility and duration of untreated psychosis: distance as a determinant of treatment delay

**DOI:** 10.1186/s12888-017-1345-8

**Published:** 2017-05-10

**Authors:** Erling Inge Kvig, Beate Brinchmann, Cathrine Moe, Steinar Nilssen, Tor Ketil Larsen, Knut Sørgaard

**Affiliations:** 1 Nordland hospital Trust, Bodø, Norway; 2 UIT The Arctic University of Norway, Tromsø, Norway; 3 Nord University, Bodø, Norway; 4 Helgeland hospital Trust, Mo i Rana, Norway; 5 Stavanger University Hospital, Stavanger, Norway; 6 University of Bergen, Bergen, Norway

**Keywords:** Dup, Treatment delay, Pathways, Accessibility, Psychosis

## Abstract

**Background:**

The duration of untreated psychosis is determined by both patient and service related factors. Few studies have considered the geographical accessibility of services in relation to treatment delay in early psychosis. To address this, we investigated whether treatment delay is co-determined by straight-line distance to hospital based specialist services in a mainly rural mental health context.

**Methods:**

A naturalistic cross-sectional study was conducted among a sample of recent onset psychosis patients in northern Norway (*n* = 62). Data on patient and service related determinants were analysed.

**Results:**

Half of the cohort had a treatment delay longer than 4.5 months. In a binary logistic regression model, straight-line distance was found to make an independent contribution to delay in which we controlled for other known risk factors.

**Conclusions:**

The determinants of treatment delay are complex. This study adds to previous studies on treatment delay by showing that the spatial location of services also makes an independent contribution. In addition, it may be that insidious onset is a more important factor in treatment delay in remote areas, as the logistical implications of specialist referral are much greater than for urban dwellers. The threshold for making a diagnosis in a remote location may therefore be higher. Strategies to reduce the duration of untreated psychosis in rural areas would benefit from improving appropriate referral by crisis services, and the detection of insidious onset of psychosis in community based specialist services.

## Background

Past research has found considerable treatment delay following a first episode of psychosis [1]. Delayed treatment leads to unnecessary distress for patients and families, and may also have long-term effects on symptom and functional outcomes [2]. Understanding the determinants of treatment delay is important for service planners and initiatives aimed at reducing duration of untreated psychosis (DUP) [3].

In early psychosis, the treatment status of patients is not a stochastic event, but is co-determined by the nature of the illness itself [4]. Treatment seeking and detection may be influenced by illness related factors such as an insidious course of illness, lack of insight or difficulties in discriminating between personality traits and illness. Specifically, numerous studies have replicated the finding that the more the three clinical factors poor premorbid function, gradual mode of onset, and adolescent onset are present, the longer may be the DUP [5–17]. While such clinical features are important determinants of DUP, other non-clinical determinants may also impact on treatment delay. Studies of what has been termed pathways to care [18] can elucidate such determinants by exploring how differences in pathways translate into differences in the DUP. This study was designed to explore to what extent DUP is co-determined by the structures of health services and the location of these services.

Changes in the structure of specialist psychiatric care after deinstitutionalization have greatly improved equity and geographical access of specialist services on average [19]. However, this gain in proximity has been accompanied by an increase in the organizational complexity of services. Specialist psychiatric care is provided by a network of local and regional services. The effectiveness of the system depends largely upon the effectiveness of referral or directing procedures.

The use of «crisis services», either accident and emergency services leading to early admission [20], or acute home treatment teams [21], have been shown to be associated with shorter DUP. It has been suggested that at least in urban settings with dense populations, admission to a 24 h emergency clinic is one of the most effective interventions for reducing DUP [20].

Most studies until now, have been conducted in settings with at least moderate population density [22]. Less is known about the effect of crisis services on DUP in settings with lower population densities. In such areas, the availability and geographical accessibility of services may vary considerably, potentially affecting the range of services used, the rate of utilization, and the timing of service use.

In the county of Nordland in North Norway, the provision of acute psychiatric care is for many patients located at a distance, at the regional general hospital. This study was designed to explore whether the routes taken and the timing in terms of DUP, is co-determined by geographical factors. The Norwegian system, in its rural configuration, offers a unique opportunity to explore the effects of the physical environment on pathways to care and treatment delay.

We conducted a naturalistic cross-sectional study of DUP and pathways to care in the northern part of Norway, a rural area where people live mainly in provincial towns or sparsely populated areas. Our aims were to (a) provide a descriptive epidemiology of the pathways to care in a mainly rural setting; (b) test the hypothesis that straight-line distance to specialist psychiatric acute wards impacts significantly on DUP, controlling for other known risk factors and pathways indicators.

## Methods

### Setting

Northern Norway is an extensive area, stretching from south of the arctic circle to the North Cape. It covers 45% of the total area of Norway, but is the home of only 10% of its population, making this one of the most sparsely populated areas in the world with an average density of 4.1/km^2^. Nordland county, one of three counties in this region, has a population of 240,000 and a population density of 7 persons per km^2^. Although there are long geographical distances between municipality centers, communications are well developed with several daily air-flights, express boats, coastal liners, and a modern system of roads.

Health care is organized as a two-level public health care system, where general practitioners (GPs) serve as gatekeepers for all specialist health services. The conventional pathway to specialist care is through the GP, but other pathways, bypassing the regular GP, exist. Importantly, emergency care is provided by regular GPs during office hours, while out-of-hours emergency care is organized by local municipalities with GPs on call, usually based in an emergency clinic. Specialized mental health care is supplied by psychiatric departments in general hospitals and community centers. Most people with common mental health problems can be referred, although persons referred for moderate or severe conditions have a right to prioritized specialist health care.

### Participants

The sample comprised consecutive patients with recent onset psychosis making contact with the central hospital or one of the community mental health centers in Nordland county. Patients were eligible for the study if they were between 15 and 35 years old, presenting with one or more positive psychotic symptoms rated by their clinician as moderate or above (4 or above) on the Positive And Negative Syndrome Scale (PANSS) [23]. Written informed consent was obtained to administer the clinical assessments, which were approved by the Regional Ethics Committee (notification 2009/1426).

Patients were recruited over a 3 year period (September 2010–September 2013). 77 patients were referred to the study, and 72 of these was asked to participate (2 patients did not meet the inclusion criteria, and 3 were discharged before they could be approached). Complete data were available on 62 (86%) patients due to drop-out and refusal on the part of the patients.

### Data collection

Participants were assessed using a battery of standardized assessments, including the Nottingham Onset Schedule-DUP version (NOS-DUP) [24], the Gater encounter form [25], the Premorbid Adjustment Scale (PAS) [26], and the OPCRIT+ checklist [27]. The NOS-DUP contains two parts: a preliminary assessment schedule completed from case notes for establishing important key dates and anchor points, and a semi-structured interview. Interview data were subsequently checked across case records, hospital records and by interviews with family informants. We also synthesized the data on pathways onto visual «route timelines», documenting the sequence of contacts, the presenting complaints of the patient, diagnosis recorded, referrals made and treatment provided [21]. Socio-demographic data, including zip code data, was based on a structured schedule. All ratings were performed by the individual investigator, and later reviewed in consensus meetings using all available data including transcripts of interviews and case notes. The three investigators (EIK, BB, CM) who carried out the assessments had completed a training and reliability program supervised by the developers of the NOS-DUP, which included the use of a modified Gater encounter form to record pathways to care and the Premorbid Adjustment Scale to record premorbid functioning [28].

The allocation of research diagnosis was done using a best-estimate consensus rating procedure utilizing the OPCRIT+ checklist [27]. The principal investigator (EIK) presented the individual assessments to an experienced psychiatrist (SN) who remained blind to the identity of the patient. All data from interviews, referral letters and case notes from medical records were available. Symptom ratings on the OPCRIT+ checklist were done individually. Any differences were resolved through discussions until consensus was achieved. The OPCRIT+ computer program generated diagnoses according to the operation criteria of 12 major classificatory systems (including DSM-IV and ICD-10).

### Outcome measures

Our main outcome measure was duration of untreated psychosis, defined as the time period between onset of psychosis and the onset of what has been termed criteria treatment [29]. The definition of these time points was as follows: (1) The onset of psychosis was defined as at least one positive symptom (as defined by the PANSS [23]) rated as moderate or above (4 or above) and lasting “throughout the day for several days or appeared several times a week, not just for a brief moment» [29] p. 246; (2) The onset of criteria treatment was defined as the date when treatment was commenced. This is defined as adhering to recommended dosage levels (defined as antipsychotic medication of 3.5 haloperidol equivalents) and continued for at least 1 month. In the case of admission to acute care, this date was used as onset of criteria treatment.

### Explanatory variables

#### Geographical accessibility

Geographical access was defined as the distance which must be travelled in order to use health services, and was operationalized as the straight line distance between patient zip code of residence to nearest health services calculated using a web based distance calculator utilizing Google Maps [30]. Zip code location data were available for all patients, and two distance variables were calculated 1) distance to specialist community services; and 2) distance to specialist psychiatric acute ward.

#### Socio-demographic variables

Standard socio-demographic information was available for all patients. Patients were classified as «not married» if they were neither married nor cohabiting at the time onset of illness. Education was classified as either greater than 10 years, or less than 10 years of state schooling. Unemployed was defined as no part- or full-time school or employment.

#### Clinical indicators

Three patient level indicators, strongly associated with DUP in previous research, were extracted from the assessment schedule. Premorbid functioning was assessed using the PAS [26]. The premorbid phase was defined as a period prior to onset of prodromal or psychotic symptoms. The PAS covers two dimensions - academic and social functioning - measured in childhood (up to 11 years) and early adolescence (12–15 years) [9]. We used the method of Larsen et al. [9] to calculate changes in functioning: PAS social change and PAS academic change scores. Change was calculated as the difference between the early adolescence score and the childhood level score. For the analysis we used a dichotomized variable for age at onset: adolescent onset (<18 years) and adult onset (>18 years). Mode of onset was defined as the speed with which psychotic symptoms emerge. Mode of onset was dichotomized as acute onset (onset definable within 1 month) versus non-acute onset (gradual onset greater than 1 month) in the analysis. In addition, diagnosis at first presentation was used in the analysis. We used a dichotomized variable for diagnosis where patients with schizophrenia and schizoaffective disorder were combined into a «schizophrenia spectrum» group, and patients with affective psychosis, brief psychosis and delusional disorder were combined into a group entitled “other psychosis”.

#### Pathways indicators

Pathways to care refers to the various help-seeking contacts made between the onset of illness and engagement in treatment [28]. A contact was defined broadly as an encounter where an individual receives an intervention, advice or referral. From the Gater encounter forms and route timeline we derived four pathway indicators:

Point of entry refers to the contact from whom help was first sought after the onset of psychotic symptoms [31]. For the analysis we classified first contacts as a) general practitioner (GP), b) emergency clinic, c) non-health agency (eg religious contacts), and d) already in specialist services. Referral source denotes the contact who suggested or arranged contact with mental health services, and was classified as referral by either a) GP, b) emergency clinic, c) self/lay referral, or d) already in specialist services. For the analysis we also extracted an acute/non-acute referral variable, defined as a) acute referral by GPs or emergency clinic, b) non-acute referral by GP, lay/self or already in services. For the analysis we classified first mental health contacts as either a) community based specialist care, and b) admission to hospital based specialist services.

### Data analysis

Preliminary analysis was performed to examine the distribution of outcome and explanatory variables. Due to a positively skewed distribution of the outcome variable, patients were divided into two groups of long and short DUP using a median split of the DUP. This dichotomization was used to compare subgroups in terms of demographics and the explanatory variables of interest. Non-parametric tests were used in bivariate analysis. All tests were two-tailed with a significant level of .05. We used a binary logistic regression model to assess the association between distance and DUP with and without referral source, alone and adjusted for the traditional risk factors and pathways indicators. Predictors were chosen on the basis of previous literature [5–17]. Even though diagnosis only approached significance in bivariate analysis, based on previous research this was considered to be an important variable, and was included in the regression model. The dependent variable was DUP, an odds ratio less than 1 indicates that as the predictor increases, the odds of a long DUP decrease, whereas an odds greater than 1 indicates that as the predictor increases, the odds of a long DUP increase. To make the odds ratio easier to interpret and more clinically meaningful, we used a transformed distance variable in the regression analysis, and odds ratio were reported per 1 standard deviation change in the distance variable. Due to a small sample size, only five independent variables were included in the models. Because we had hypothesis for most comparisons no adjustment for multiple testing was employed. The interaction effect between distance and acute referral, calculated by the product of the two variables, was non-significant. The final model was checked for violations of assumption, the effect of outliers and influential observations. Data were analyzed using SPSS (version 21) for Macintosh.

## Results

A summary of the sample characteristics (*n* = 62) is presented in Table 1. The sample comprised 44 (71%) male and 18 (29%) female patients, with a mean age of onset of 19.9 years (s.d. = 4.2). The majority of patients were not married, had less than 10 years of education, and were unemployed at the time of onset. The OPCRIT diagnoses included schizophrenia (77.4%), schizoaffective disorder (1.6%), affective psychosis (1.6%), brief psychosis (17.7%) and delusional disorder (1.6%).

**Table 1:** Socio-demographic, clinical and pathways indicators (*n* = 62)

Category	Number with characteristics from whole cohort
Socio-demographic variables (n (%))	
Male	44 (71.0)
Not married	60 (96.8)
Education (< 10 years)	39 (62.9)
Unemployed	43 (69.4)
Diagnostic categories (n (%))	
Schizophrenia diagnosis	49 (79.0)
Other psychosis	13 (21.0)
Premorbid and onset parameters (n (%))	
Non-acute mode of onset, >1 month	36 (58.0)
Adolescent onset	20 (32.3)
Premorbid social change (mean (range))	.0403 (−2.5–2.5)
Specialist referral source (n (%))	
Acute referral (emergency, GP, police)	18 (29.0)
Non-acute referral (GP, lay/self, already in services)	44 (71.0)
First mental health contact (n (%))	
Admission to specialist psychiatric acute ward	25 (40.3)
Community based specialist care	37 (59.7)
Geographical accessibility in kilometers (mean (range))	
Distance to specialist community care	19.9 (0–69)
Distance to specialist psychiatric acute ward	99.32 (3–241)

### Distance variables

Distances to specialist psychiatric care were long for both access measures. The mean straight-line distance to nearest community care centre was 19.9 km, with a maximum of 69 km. This corresponded to an estimated 43 min and 149 min travel time. The mean straight-line distance to the psychiatric acute ward located at the regional central hospital was 99.3 km, with a maximum of 241 km. Corresponding travel times was 4.3 h and 11 h. In terms of rurality, 33 patients (53.2%) lived in a rural areas with a population less than 10,000 people, and 29 (46.8%) patients lived in provincial towns with populations between 10,000 and 100,000 people.

### Dup

For the complete cohort (*n* = 62) median DUP was recorded at 18.5 weeks (IQR: 4–59.75), with a mean of 77 weeks (Table 2). Patients in the long DUP group had a median of 57 weeks (mean 147.7, 173.7 s.d.), while the short DUP group had a median of 4 weeks (mean 5.9, 35.9 s.d.).

**Table 2:** Duration of Untreated Psychosis (DUP) and delay variables

	Mean	SD	Median	IQR	Min - Max
Age at onset (years)	19.9	4.2	19	17.0–22.3	12–33
Duration of untreated psychosis (weeks)	76.8	141.3	18.5	4.0–59.8	0–693
Duration of prodrome (weeks)	129.1	121.2	95.5	23.8–206.0	0–626
Duration of untreated illness (weeks)	206.3	186.9	163.0	63.8–326.0	2–797

### Socio-demographic, clinical and pathways correlates of DUP

Table 3 compares demographic characteristics, clinical and pathways indicators in patients with short and long DUP. There were no significant differences between subgroups of DUP on demographic variables such as age, gender, education, employment or marital status. Significant differences were present for only one of the clinical indicators: mode of onset. When grouped according to mode of onset, 26 (42%) patients had an acute onset of the psychotic episode and 36 (58%) patients had a non-acute onset. There was a significant group difference in the presence of a non-acute onset of psychosis (Chi Χ^2^ (1) = 9.538, *p* = .004). Specifically, patients in the long DUP group were more likely to have a non-acute onset of psychosis than the short DUP group. Non-acute onset was significantly related to both delayed help-seeking (Mann-Whitney, U = 610, z = 2.15, *p* = .031) and treatment delay after being referred to mental health services (Mann-Whitney, U = 610, z = 2.15, *p* = .035). There were no significant differences between subgroups of DUP on age at onset or premorbid functioning.

**Table 3:** Comparison of socio-demographic, clinical and pathways indicators in patients with short vs long DUP (*n* = 62)

Category	Short DUP	Long DUP	*P* ^a^
Socio-demographic variables (n (%))			
Male	22 (35.5)	22 (35.5)	1.0
Not married	31 (50.0)	29 (46.8)	.492
Education (< 10 years)	17 (27.4)	22 (35.5)	.324
Unemployed	19 (30.6)	23 (37.0)	.416
Diagnostic categories (n (%))			
Schizophrenia diagnosis	21 (33.9)	28 (45.2)	.059
Premorbid and onset parameters (n (%))			
Non-acute mode of onset	12 (19.4)	24 (38.7)	.004
Adolescent onset	8 (12.9)	12 (19.4)	.416
First contact (n (%))			
General practitioner	10 (16.1)	14 (22.6)	
Emergency clinic	9 (14.5)	5 (8.0)	
Non-health contact	4 (6.5)	4 (6.5)	
Already in specialist services	8 (12.9)	8 (12.9)	.616
Specialist referral source (n (%))			
Acute referral (emergency clinic, GP)	14 (22.6)	3 (4.8)	
Non-acute referral (GP, lay/self, already in services)	16 (25.8)	28 (45.2)	.002
First mental health contact (n (%))			
Admission to hospital services	17 (27.4)	8 (12.9)	
Community based specialist care	14 (22.6)	22 (35.5)	.037
Geographical accessibility in kilometers (mean (median))			
Distance to specialist community care	21.6 (14)	18.4 (14)	.569
Distance to specialist psychiatric acute ward	78.2 (43)	120.4 (144)	.044

^a^The X^2^ test was used for categorical variables and the Kruskal-Wallis test for variables with multiple categories

**Fig. 1:**
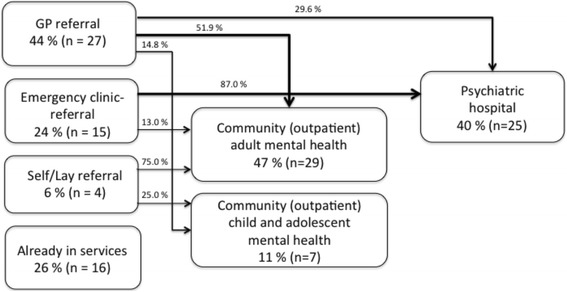
Pathways diagram for 62 patients in Nordland county. Referral source ratios and following steps of the pathway to specialist mental health services are presented. The figures show the percentage and number of subjects who took each pathway

Three pathways indicators were examined in relation to subgroups of DUP. First contacts and referral patterns are presented in Fig. 1. There was no significant difference in DUP according to point of entry into services. A high level of GP involvement in referral was expected as a consequence of their gatekeeping function, but emergency clinic involvement anywhere on the pathway was unexpectedly high. For 15 patients (24.2%) contact with an emergency clinic led to a specialist referral, while a total of 23 patients (37.1%) had contact with an emergency clinic anywhere on their pathway. Acute referral was more common in the short DUP group (Chi Χ^2^ (1) =11.27, *p* = .002). Furthermore, the association between acute referral and an acute mode of onset was significant (Chi Χ^2^ (1) = 8.011, *p* = .007).

First contact with specialist services comprised two groups: (1) admission to hospital, *n* = 25 (40.3%) and (2) community based specialist care for adults (CMHC), *n* = 29, 46.8%, and children and adolescents (CAMHC), *n* = 7, 11.3%. One patient received criteria treatment from his GP without specialist referral. Among the 16 patients already receiving treatment in specialist care at the time of psychosis onset, 12 (19.4%) patients were in community specialist care, and 4 (6.5%) patients were in hospital-based specialist care. There was a statistically significant difference between subgroups of DUP and first mental health contact. Patients admitted at first contact were more likely to have a short DUP than patients receiving community care at their first mental health contact.

### Binary logistic regression analysis

The majority of patients received criteria treatment after admission (*n* = 44, 71%), and longer straight-line distance to specialist psychiatric acute care was significantly related to long DUP in bivariate analysis (Mann-Whitney U = 622, z = 2.01, *p* = .044). Only distance to specialist psychiatric acute care was therefore examined in the regression models.

As decisions made by referral agents may have important distance modifying effects, particularly the decision of acute vs non-acute referral, the unadjusted and adjusted odds ratios for these two variables crudely associated with DUP is shown in Table 4. The unadjusted odds ratios showed a crude association between DUP and the distance variable, and a strong crude association between DUP and non-acute referral. In model 2 we entered these two variables together and they retained independent contribution to DUP, with odds ratios of 2.1 and 7.69, respectively. This model predicted long DUP correctly in 77.4% of the patients, while short DUP was predicted correctly in 71% of the patients. Overall, the outcome was predicted correctly for 74.2% of the patients by the model. Risk estimates were only slightly attenuated when including other known risk factors for long DUP (diagnosis and mode of onset) and the variable admission as first mental health contact.

**Table 4:** Binary logistic regression models with long vs short duration of untreated psychosis as dependent variable

	Crude		Model 2		Model 3^a^	
	OR (95% CI)	*P* Value	OR (95% CI)	*P* Value	OR (95% CI)	*P* Value
Distance to acute wards^b^	1.83 (1.07–3.14)	.027	2.14 (1.15–3.98)	.016	2.09 (1.04–4.18)	.037
Non-acute referral	8.750 (2.19–34.9)	.002	11.22 (2.53–49.72)	.001	11.00 (1.79–67.74)	.010

R^2^ = .33 (Hosmer & Lemeshow) .37 (Cox & Snell).49 (Nagelkerke). Model: Chi Χ^2^(5) = 28.539, *p* < .000

^a^Adjusted for non-acute mode of onset, schizophrenia diagnosis, first mental health contact: non-admission

^b^ORs per 1 standard deviation change in distance variable

## Discussion

In Norway, the highly successful Scandinavian TIPS (early Treatment and Intervention in Psychosis) project [32], has had a great impact on service availability and awareness of early psychosis among both the lay public and professionals in the public health system. The finding of a median DUP of 18.5 weeks in the current study, indicating that half of the cohort received adequate treatment within 4–5 months, is well above the national average of 9.7 weeks (mean 67.7 weeks) reported in a recent study [33]. The common finding of a positively skewed distribution of DUP was also found in this study, indicating that the mean is inflated by a cohort of patients with very long DUP. Using multivariable logistic regression analysis, we found support for the hypothesis that distance to psychiatric acute wards has an independent effect on long DUP. The effect of geographical accessibility, in terms of straight line distance, remained significant after adjusting for risk factors such as schizophrenia diagnosis and non-acute mode of onset, and for pathways indicators such as non-acute referral and non-admission at first mental health contact.

We have replicated previous studies showing that mode of illness onset is a reliable illness related determinant of DUP [13, 29, 34, 35]. Consistent with other recent studies we found that use of «crisis services» is the most rapid and effective pathway to care [36, 37, 21, 20]. Acute referral to specialist mental health care occurred in 27% in our sample, consistent with the figures reported in other studies [36, 20]. The finding that 37% of our sample had at least one contact with the emergency clinic on their pathway was surprising, and indicates that for a number of patients a emergency contact did not translate into an appropriate psychiatric referral or initiation of adequate treatment. It is important to underline that in all these cases the patients were actively psychotic and untreated. This is however consistent with studies of Norwegian emergency clinics, reporting that four out of five patients presenting with mental illness are managed without hospital referral [38]. These results suggests that more studies are needed on the appropriateness of referral decisions by emergency clinics. However, service entry for the majority of patients in this study sample was through non-crisis agencies. Several studies have confirmed treatment delay within mental health services, and particularly first contact with generic mental health services predicts substantial delay [15, 21, 36]. Our finding that patients who are already in contact with specialist services at the time of psychosis onset, often experience long DUP is consistent with the findings in other studies [36].

To our knowledge there have been no previous studies on the relationship between distance to health care services and DUP. Some studies have however reported on rural-urban comparisons in relations to DUP, but with inconsistent results [36, 39–41]. Other pathways to care studies have found that rural citizens generally have more contacts with traditional healers, GPs or primary health carers before they enter specialist mental health care [42–45]. Several studies have documented distance effects on utilization rates. The early study by Edward Jarvis was the first to document that people living near psychiatric hospital send more patients there for admission than do those living far away [46]. Later studies have replicated these findings, and found support for the so-called «Jarvis law» or «distance decay model» in mental health care [47–51]. Distance decay effects have also been found in utilization rates of out-of-hours causality clinics [52] and referral rates to hospitals [53]. A common finding in previous studies on service utilization is that severity of illness and an effective referral systems can act as modifiers of distance effects [54]. Our results indicate that in first episode psychosis, with great heterogeneity in clinical presentations, psychotic patients with milder symptom profiles could still be at greater risk of treatment delay. In patients with obvious and visible psychotic symptoms the imperative need for treatment is probably readily recognized regardless of distance to appropriate specialist services. This «sense of urgency» may not be evoked in patients with a more non-acute onset, and a decision to refer will be heavily influenced by the perceived treatment gain in relation to the costs of sending the patient at great distances to a psychiatric acute ward.

This study, which to our knowledge is the first to report on distance as a determinant of DUP, has important strengths. How the physical context impact on the social process of help-seeking and service responses, is under-researched in studies of determinants of DUP. The setting of this study, a large area with a great variety of distances, makes it well suited to study how geography influences treatment delay in first episode psychosis. Socioeconomic and demographic similarities between catchments areas, absence of private service providers, and the overarching national standards of a public health system, helps to rule out confounding variables in the interpretation of the results. The findings will have relevance nationally and internationally, given that spatial location of health services are important in the landscape of care in many countries.

However there are some limitations to the study. Our results were based on a small sample size which limited the number of variables we could include in multivariable analysis. A larger sample would allow a more detailed examination of other variables such as ethnicity, social deprivation or clinical variables such as symptom severity and positive vs negative symptoms. In addition, although we used structured research instruments and trained raters, DUP by definition requires a retrospective account of symptomatology by the patients. This can lead to recall bias. In this study the potential influence of recall bias was reduced by conducting interviews after antipsychotic treatment had been initiated, and cross-checking with information on symptomatology and treatment contacts obtained from family informants and medical files.

Efforts to reduce DUP need to be informed by a framework on pathways to care that recognizes that the determinants of treatment delay are multifaceted, and likely a result of an interplay of illness related and contextual factors, and where the impact of patient factors may vary depending on the specific context. In early psychosis, the mentally ill person may neither be able to recognize the existence of illness nor to evaluate different treatment alternatives, placing the person in the mercy of the clinical decision-making and referral behavior of health care professionals. In real-world settings this process will be influenced both by clinical and non-clinical factors. We suggests that in rural settings, estimation of spatial separation, or «cognitive distance» [55], can influence clinical decision making process and potentially delay treatment. Distance effects are perhaps more likely in in patients with non-acute onset where the sense of urgency naturally evoked by a more acute onset is absent.

In sparsely populated areas strategies to reduce DUP would benefit from increasing the effectiveness of the health systems referral system. In the Norwegian public health system, improving appropriate referral through the already established and effective crisis services, including GP, emergency clinics, and psychiatric acute wards, would be an important target. In addition, strategies to improve the detection rate of insidious cases and emphasize a similar «sense of urgency» in these cases could be effective in reducing DUP. As many patients are already in treatment with their GP or community mental health centers at the time of onset, enhancing knowledge of insidious features of psychosis in theses settings would be a viable option.

## Abbreviations

CIs: Conficence intervals
DSM-IV: American Psychiatric Association Diagnostic and Statistical Manual of Mental Disorders, fourth edition
DUP: Duration of untreated psychosis
GP: General practitioner
ICD-10: International Statistical Classification of Diseases and Related Health Problems, 10th revision
NOS-DUP: Nottingham Onset Schedule - DUP version
OPCRIT +: Operational Criteria checklist, enhanced version
OR: Odds ratio
PANSS: Positive and Negative Syndrome Scale
PAS: Premorbid Adjustment Scale
SPSS: Statistical Package for the Social Sciences
TIPS: Early Treatment and Intervention in Psychosis
